# Quantitative Cortex‐Based Mapping With Hybrid ^18^F‐FDG‐PET/MR Images in MRI‐Negative Epilepsy

**DOI:** 10.1111/cns.70336

**Published:** 2025-04-18

**Authors:** Chao Zhang, Zhenming Wang, Yihe Wang, Hang Cao, Liankun Ren, Tao Yu, Yong‐Zhi Shan, Xiaosong He, John S. Duncan, Jie Lu, Penghu Wei, Guoguang Zhao

**Affiliations:** ^1^ Department of Neurosurgery, Xuanwu Hospital Capital Medical University Beijing China; ^2^ Clinical Research Center for Epilepsy Capital Medical University Beijing China; ^3^ Beijing Municipal Geriatric Medical Research Center Beijing China; ^4^ Department of Radiology and Nuclear Medicine, Xuanwu Hospital Capital Medical University Beijing China; ^5^ Department of Radiation Oncology, Xuanwu Hospital Capital Medical University Beijing China; ^6^ Department of Clinical & Experimental Epilepsy UCL Queen Square Institute of Neurology London UK; ^7^ Department of Neurology, Xuanwu Hospital Capital Medical University Beijing China; ^8^ Beijing Institute of Functional Neurosurgery, Xuanwu Hospital Capital Medical University Beijing China; ^9^ Department of Psychology University of Science and Technology of China Hefei Anhui China; ^10^ MRI Unit Chalfont Centre for Epilepsy Gerrards Cross UK

**Keywords:** evaluation, neurosurgery, outcomes, postprocessing, seizures

## Abstract

**Objectives:**

Localization of the network underlying drug‐resistant focal epilepsy in individuals considering surgical treatment with unremarkable MRI is challenging. Concordance rates of 40%–69% have been reported with FDG‐PET image statistical parametric mapping (SPM). We investigated the efficacy of postprocessing specific to cortices by cortex‐based mapping (CBM) on hybrid PET/MR images with healthy subjects to localize sites of seizure onset.

**Methods:**

We retrospectively examined the PET/MR images of 42 MRI‐negative individuals with drug‐resistant focal epilepsy who had surgery and 23 healthy subjects. Visual interpretation of standardized uptake value ratios (SUVRs), voxelwise mapping with a two‐sample *t*‐test of SUVRs (t‐map, SPM), and the proposed z‐transformation of the SUVR of patients compared with those of healthy subjects acquired with CBM were compared with the surgical field. Kappa tests, conclusive concordance (CC), partial concordance (PC), and discordance were estimated, with McNemar's test determining the superiority.

**Results:**

After an average follow‐up of 37.2 months, in people who were seizure‐free (*n* = 31; functionally silent cortices in 26), the CC rate with CBM was 87.10%. Performance was CBM (CC:PC = 27:1), t‐map (CC:PC = 15:1), and SUVR (CC:PC = 0:17). The sensitivity, specificity, and kappa scores were 0.87, 0.91, and 0.717 (*p* < 0.001) for CBM and 0.48, 0.73, and 0.153 (*p* = 0.288) for t‐maps, respectively. The CBM approach was superior to the t‐map (*p* < 0.001) in most extratemporal epilepsies. The average Pearson's *r* of CBM and t‐map to artifacts was 0.08 ± 0.02 and 0.33 ± 0.02, respectively.

**Interpretation:**

By eliminating intersubject morphological variations and explicit statistics at the cortex, CBM localized the seizure origin in MRI‐negative epilepsy patients with superior efficiency.

## Graphical abstract

Cortex‐based mapping (CBM) with hybrid PET/MR images effectively localizes seizure origins in MRI‐negative epilepsy patients, primarily in functionally silent cortices. This study introduces a refined protocol and demonstrates that, by focusing on cortical statistics, CBM offers a significant advancement in presurgical evaluation for patients with atypical seizures.

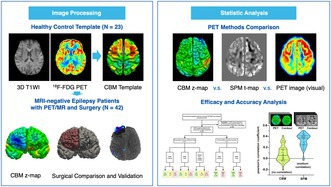

## 1 Introduction

Compared to focal epilepsy with identifiable lesions, the success rate of surgery for MRI‐negative epilepsy is lower [1, 2]. Up to 90% of individuals with MRI‐negative drug‐resistant focal epilepsy (DRFE) may be considered candidates for surgical intervention, which offers significant potential for improving seizure control [3, 4]. Improvements in seizure outcomes depend on increased accuracy of localization of the seizure onset zone. Semiology may be insensitive or specific, particularly if seizure onset is in a silent cortex [5, 6, 7], and the epileptogenic zone may be only a 13 × 7 × 6 mm region [8]. Scalp electroencephalography (EEG) only detects abnormalities extending to over 6 cm^2^ on the cortical surface and is inconclusive for deep cortical regions [9, 10]. Intracranial EEG has limited coverage, and the earliest epileptic discharge detected might be due to seizure propagation rather than indicating the site of seizure onset [3, 7]. Thus, seizure localization in MRI‐negative individuals with DRFE is challenging.

^18^F‐FDG PET has been used for seizure localization, with visual identification of focal hypometabolism [11, 12]. Coregistration of PET images to MRI enhances the evaluation [13, 14]. However, non‐epileptic brain areas may exhibit hypometabolism, and in rare instances, the epileptogenic zone might show hypermetabolism [15, 16]. Quantitative methods for PET imaging have been developed to enhance sensitivity and specificity and to improve clinical outcomes [17]. Determining the asymmetry of glucose uptake in regions of interest (ROIs) may be helpful in some cases, but does not reliably detect small areas of abnormality [18]. Voxel‐based analysis of FDG PET may detect focal hypometabolism in 40%–69% [19, 20, 21]. In people with inconclusive visual interpretations of routine PET images, the detection rate was 40% [19], indicating that limitations remain. For example, an accurate overlap of the gyrus or sulcus between participants may not be achieved with volume‐based intersubject coregistration methods, limiting the ability to compare regions uniformly across individuals [22]. Patients with MRI‐negative DRFE typically exhibit cortical abnormalities [23], and the inclusion of white matter signals during smoothing procedures decreases accuracy.

Hybrid PET/MRI has been suggested to be helpful for localizing abnormalities in those with DRFE [24, 25]. The structural MR images obtained during ^18^F‐FDG PET/MR scanning enable detailed coregistration and spatial segmentation [26]. The cortex‐based method combining volumetric and surface registration approaches provides more accurate metabolic detection and uniform comparisons across subjects [22, 27]. However, the effectiveness of cortex‐based registration and segmentation for localizing epileptogenic foci in DRFE with FDG‐PET/MRI images has not been fully determined. An ^18^F‐FDG PET/MR template based on healthy subjects for seizure localization has also not been fully quantified. We have validated the application of cortex‐based mapping (CBM) to FDG‐PET data acquired with hybrid PET‐MRI for identifying epileptogenic foci in a group with DRFE and unremarkable MRI.

## 2 Materials and Methods

### 2.1 Participants

Sixty‐five participants were enrolled, comprising 23 healthy subjects who had PET/MRI scans during a health examination or underwent PET scans for reasons other than the neurological referral, and 42 people with MRI‐negative DRFE. Participants in the healthy control group were screened to exclude those with any brain abnormalities, systemic conditions, or neurological disorders (Table 1). Patients' eligibility for epilepsy surgery was determined by intracranial EEG and multidisciplinary team discussions (MDT). No seizures occurred in the 4  h before the PET/MR acquisition to avoid the influence of seizure‐related brain metabolic changes on the accuracy of the imaging data [28]. Visual interpretation of the PET images, statistical parametric mapping (SPM) t‐map, and the z‐map with CBM were compared with the surgical field. The Ethical Committee of Xuanwu Hospital, Capital Medical University, approved this study. Written informed consent was obtained from all participants.

**TABLE 1:** Demographic and clinical features of the population.

Characteristics	Epilepsy patients	HCs
Total (*N*)	42	23
Median age, ranges (years)	29.0 (14–48)	31.0 (22–45)
Follow up (month)	37.19 ± 5.15	—
Sex (*N*)		
Male	26	9
Female	16	14
Age at epilepsy onset (years, mean ± SD)	11.49 ± 7.02	—
Epilepsy duration (years, mean ± SD)	17.96 ± 10.42	—
Seizure frequency (*N*)		
Monthly	12	
Weekly	13	
Daily	17	

Abbreviations: HCs, healthy controls; SD, standard deviation.

### 2.2 Data Acquisition and Partial Volume Correction

The data were collected from an integrated simultaneous time‐of‐flight (ToF) PET/MRI (Signa PET/MRI, GE Healthcare, WI, USA) system using a 19‐channel head and neck union coil. Patients were positioned supine in a low‐light environment within the PET/MR scanner room and instructed to remain relaxed with their eyes closed. The acquisition protocol for the PET/MR data and 3D T1 data was the same as in previous studies [29, 30]. A sagittal 3D (3D BRAVO) T1‐weighted sequence was acquired for registration and segmentation. The following parameters were used: repetition time, 8.5 ms; echo time, 3.2 ms; voxel size, 1 × 1 × 1 mm^3^; 178 slices.

PET data were collected in 3D list mode. MRI‐based PET attenuation was performed with an 18‐s 2‐point Dixon scan, field of view: 350 × 350 mm; matrix: 192 × 192; voxel size: 1.82 × 1.82 × 2.78 mm; and spatial resolution of 4.5 mm. Images were reconstructed with an algorithm using ordered‐subset expectation maximization. The parameters were as follows: iterations = 8; subsets = 32; and full‐width at half‐maximum (FWHM) of a 3.0‐mm Gaussian filter. Partial volume correction (PVC) was achieved by using iterative reblurring with a van Cittert method (http://www.turkupetcenter.net/tpcclib‐doc/md_install.html). The FWHM in the XY plane and in the *Z*‐axis was set to 4.5 mm. The PET images were converted into the standardized uptake value ratio (SUVR) relative to the cerebellum.

### 2.3 Voxelwise Statistical Analysis With SPM t‐Maps

SPM t‐maps are widely used in studies of seizure localization. We compared this approach with the CBM approach, using SPM12 (http://www.fil.ion.ucl.ac.uk/spm/softwar‐e/spm12). The T1 images were segmented into gray matter, white matter, and cerebrospinal fluid (CSF) using SPM12. Subsequently, the PET images were registered to the segmented T1 images and normalized to MNI152 space. The PET images were registered to the T1 images and normalized to MNI152 space. The preprocessed images were smoothed with a FWHM value of 6.0 mm. A voxelwise *t*‐test (*p* < 0.001, 20 voxels; *p* < 0.05, 20 voxels or the global maximum *t* values, depending on whether the preceding threshold was conclusive) between an individual and healthy subjects at the group level was performed. As the t‐map images were in the group level's standard space, we reverse‐normalized the t‐map back to the individual's T1 space to match clinical requirements.

### 2.4 Vertexwise Statistical Analysis With CBM Z‐Maps

CBM enables the elimination of intersubject spatial differences and statistical metabolic values in the cortex from the 3D space directly (with the smallest element called the vertex). We statistically analyzed the *z* score derived from the CBM approach. The SUVRs of the healthy participants were projected to the same average space in FreeSurfer to create a 3D template. The command “recon‐all” in FreeSurfer software (https://surfer.nmr.mgh.harvard.edu/fswiki/Dow‐nloadAndInstall) was used to segment the T1 image data. Segmentation accuracy was checked by carefully reviewing the surface boundaries in 2D space. The PVC PET images were registered to the FreeSurfer space and smoothed within the 3D surface with an FWHM value of 20. The data were compared in the average space using “freesurferformats” (https://github.com/dfsp‐spirit/freesurferformats) in R‐studio (https://www.rstudio.com/products/rstudio/download/), in which a z‐transform of the normalized SUVR at the surface vertices for each individual was performed with reference to the mean and standard deviation calculated from the healthy 3D template, and the *z* score of the SUVR at the vertices was generated to constitute the z‐maps. The global minimum 0.5% of the z‐maps was thresholded as the candidate for identification of an area of focal hypometabolism. Finally, the z‐maps were interpolated back to the corresponding surface vertices as a surface file and visualized in the individual's native space.

### 2.5 Quantifying the Intersubject Registration of SPM T‐Maps and CBM Z‐Maps

The registration step is crucial for accurately calculating intersubject statistics. Accurate overlapping of the edges in the gyri and sulci of different participants via a traditional normalization approach is still challenging. We created a model for all participants by replacing each of the gray matter intensities with a value of one and each of the white matter intensities with a value of minus one; thus, this model contained only morphological information for each individual. Each of the steps used for the SPM t‐maps and CBM z‐maps was replicated using this model to obtain quantified artifacts regarding the intersubject registration for each participant. The degree of similarity between the results of the PET data and the morphological model based on each method was calculated with Pearson's correlation analysis.

### 2.6 Comparison of the Clinical PET Images, SPM t‐Maps, and CBM Z‐Maps

We compared the outcomes of visual reading and voxelwise statistics with t‐maps with those of the CBM Z‐maps. The surgical field revealed by postoperative MR or CT images, intracranial EEG, and postsurgical seizure outcomes judged by the Engel classification system was adopted to evaluate the accuracy of the method. Seizure outcomes were categorized at least 1 year after surgery by the Engel Epilepsy Surgery Outcome Scale. Surgical outcomes were then categorized as Engel IA (seizure‐free) and Engel II–IV (non‐seizure‐free).

We assessed the statistical differences between CBM and SPM using McNemar's test for paired data, which compares the classification results of both methods. A *p* value < 0.05 was considered statistically significant. Kappa statistics were used to measure the agreement between the two methods, with values interpreted as substantial or almost perfect agreement. For continuous data, we used paired *t*‐tests to compare mean values between CBM and SPM, with a significance level of *p* < 0.05. For the calculation of sensitivity and specificity, true positives (TPs), true negatives (TNs), false positives (FPs), and false negatives (FNs) were defined as follows: TP: PET results were surgically removed and seizure‐free; FP: PET results were surgically removed but not seizure‐free; FN: The patients were seizure‐free but PET results were not surgically removed; TN: The patients were not seizure‐free and PET results were not surgically removed. Furthermore, sensitivity = TP/TP + FN, specificity = TN/TN + FP.

For people who were seizure‐free, three different situations were observed to evaluate the effectiveness of the imaging methodology. (1) Conclusive concordant (CC): the seizure origin was localized with an imaging map without needing a secondary modality. The clinician or radiologist did not need much discussion to localize the foci. (2) Partial concordance (PC): the seizure origin was localized with an imaging map only when supplemented by a secondary modality, for example, EEG or magnetoencephalography. After the clinician's or radiologist's comprehensive discussions, faulty localization is still possible. (3) Discordance: the seizure origin was not localized by the imaging map, even when supplemented by an additional modality, for example, resection of the region overlapped with the imaging map but still had seizures or identified an area outside of the resected region in a seizure‐free patient.

## 3 Results

### 3.1 Metabolism in Healthy Subjects

**FIGURE 1:**
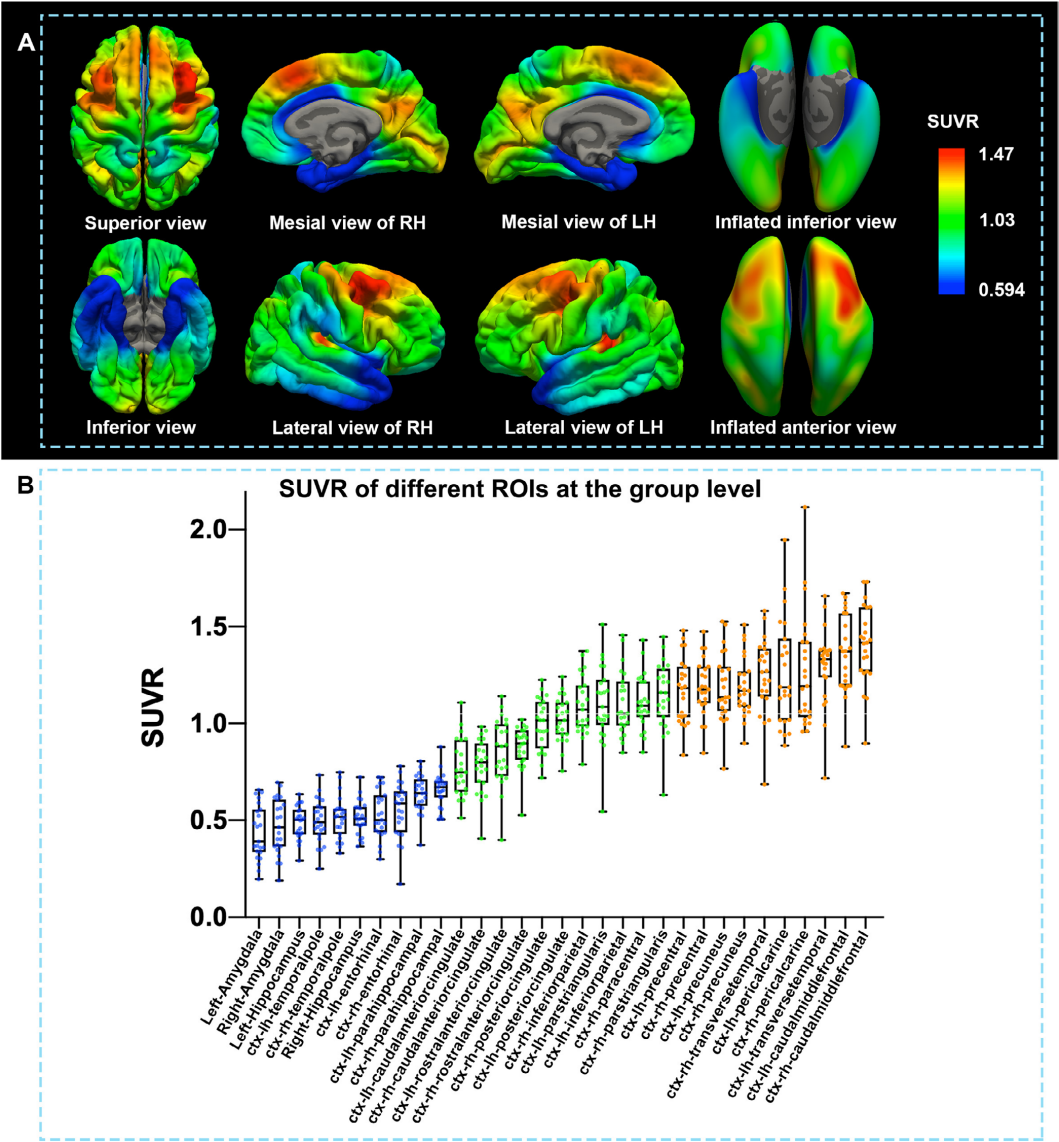
Metabolism in healthy participants revealed by SUVR. With FreeSurfer segmentation, we quantified the mean ^18^F‐FDG SUVR across different brain regions in healthy people. Note the relative areas of hypometabolism, where the SUVRs were less than those in the cerebellar cortices: Amygdala (L: 0.44, R: 0.47), temporal pole (L: 0.50, R: 0.51), and entorhinal (L: 0.53, R: 0.55), parahippocampal (L: 0.64, R: 0.66) and hippocampal (L: 0.49, R: 0.52) regions.

The SUVRs of controls are shown in Figure 1. Among controls, the dorsal middle frontal cortices had the highest mean SUVR, followed in descending order by the auditory, visual, and precentral cortices. In contrast, physiologically hypometabolic regions, where the SUVRs were less than those in the cerebellar cortices, were observed in the amygdala (L: 0.44, R: 0.47), temporal pole (L: 0.50, R: 0.51), and entorhinal (L: 0.53, R: 0.55), parahippocampal (L: 0.64, R: 0.66), and hippocampal regions (L: 0.49, R: 0.52).

### 3.2 Diagnostic Performance of Different Analytical Methods

For the 42 individuals with epilepsy, after an average follow‐up of 37.19 ± 5.15 months, routine visual assessment with only the clinical PET images (SUVR) could not reach a CC in any of them. The data from the CBM z‐map and the SPM t‐map were mainly compared with the surgical field (Table 2). The sensitivity and specificity of CBM were 0.87 and 0.91, respectively, while those of the SPM were 0.484 and 0.727, respectively. The positive predictive value (PPV) and negative predictive value (NPV) were 0.964 and 0.714, respectively, for CBM, and 0.833 and 0.333, respectively, for SPM. Accordingly, the kappa scores between the CBM or t‐map outcomes and the surgical outcomes were 0.717 (*p* < 0.001) and 0.153 (*p* = 0.288), respectively (surgical field location and results of CBM and SPM quantification, as well as the visual interpretation of routine PET images, are shown in Table S1).

**TABLE 2:** Diagnostic performance of different analytical methods.

	Sensitivity (95% CI)	Specificity (95% CI)	PPV (95% CI)	NPV (95% CI)	Kappa Score	*p*
CBM z‐map	0.871 (0.692–0.958)	0.909 (0.571–0.995)	0.964 (0.798–0.998)	0.714 (0.420–0.904)	0.717	< 0.001
SPM t‐map	0.484 (0.306–0.666)	0.727 (0.393–0.927)	0.833 (0.577–0.956)	0.333 (0.164–0.553)	0.153	0.288
SUVR (visual)	0 (0–0.137)	0.909 (0.571–0.995)	0 (0–0.945)	0.244 (0.129–0.406)	−0.048	0.594

Abbreviations: NPV, negative predictive value; PPV, positive predictive value.

### 3.3 Concordance and Discrepancies in Seizure Outcomes

**FIGURE 2:**
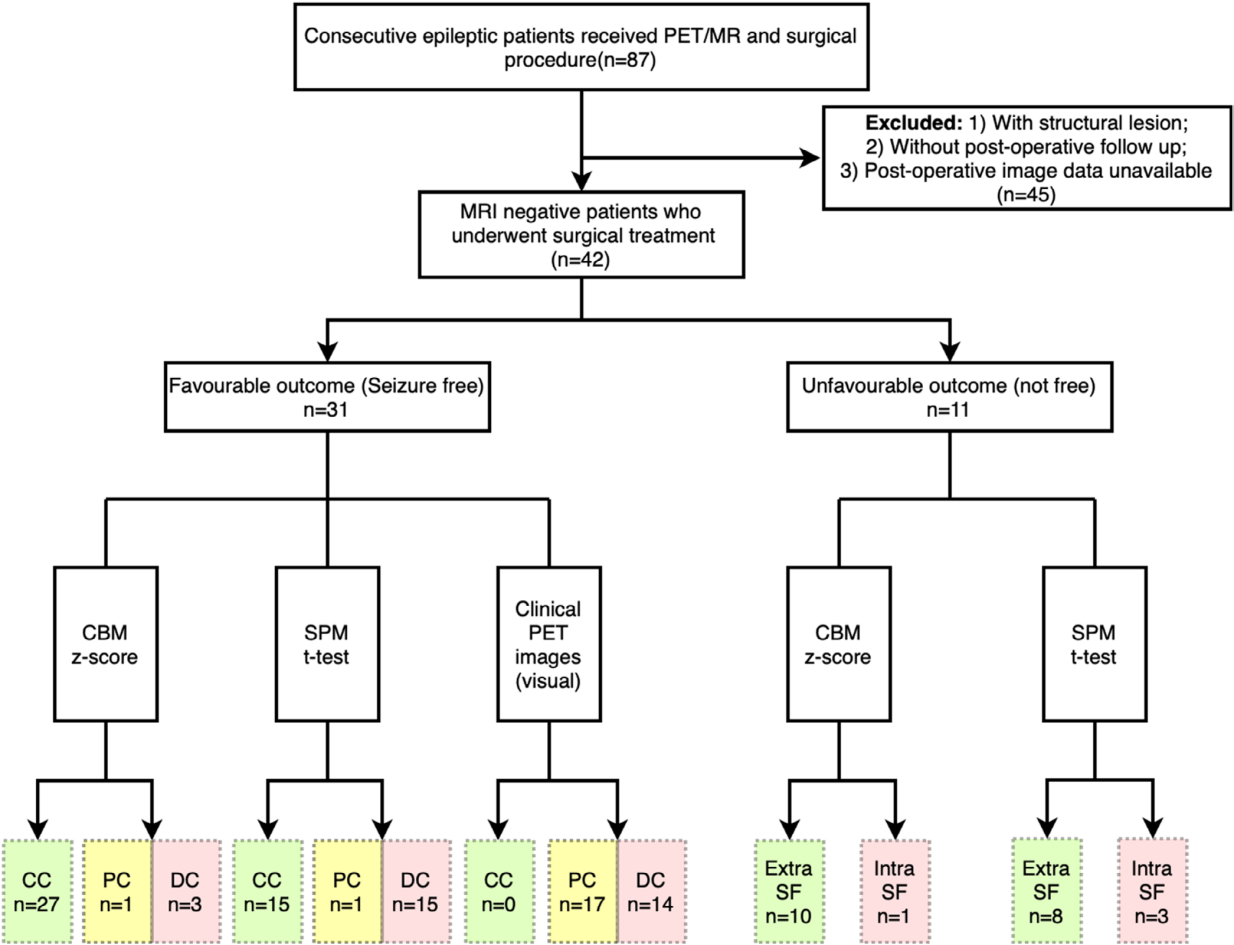
Flowcharts of case enrollment. CC, conclusive concordant; DC, discordance; Extra SF, mapping results are outside the surgical field; Intra SF, mapping results are within the surgical field; PC, partial concordance. Green color = correct results; and red/yellow color = false results.

Thirty‐one patients were seizure‐free (26 in silent cortices); they were selected as the subgroup whose epileptogenic zone was certain. We conducted a comparison of localization concordance, with results shown in Figure 2. Relative to the surgical field, CC was reached in 27 (87.10%) and 15 (48.39%) of the patients with CBM and the t‐maps, respectively (*p* < 0.001, by McNemar test) (Figure 2). When comparing the CBM results and the t‐maps concerning locations (CC/total for CBM and t‐maps), the t‐maps localized the epileptogenic zone with comparable performance for temporal lobe epilepsy (7/9, 6/9), and CBM was more effective for extratemporal epilepsies, including frontal lobe (11/12, 5/12), parieto‐occipital (3/3, 2/3), perisylvian or insular (6/6, 2/6), and parasagittal epilepsies (3/3, 1/3).

**FIGURE 3:**
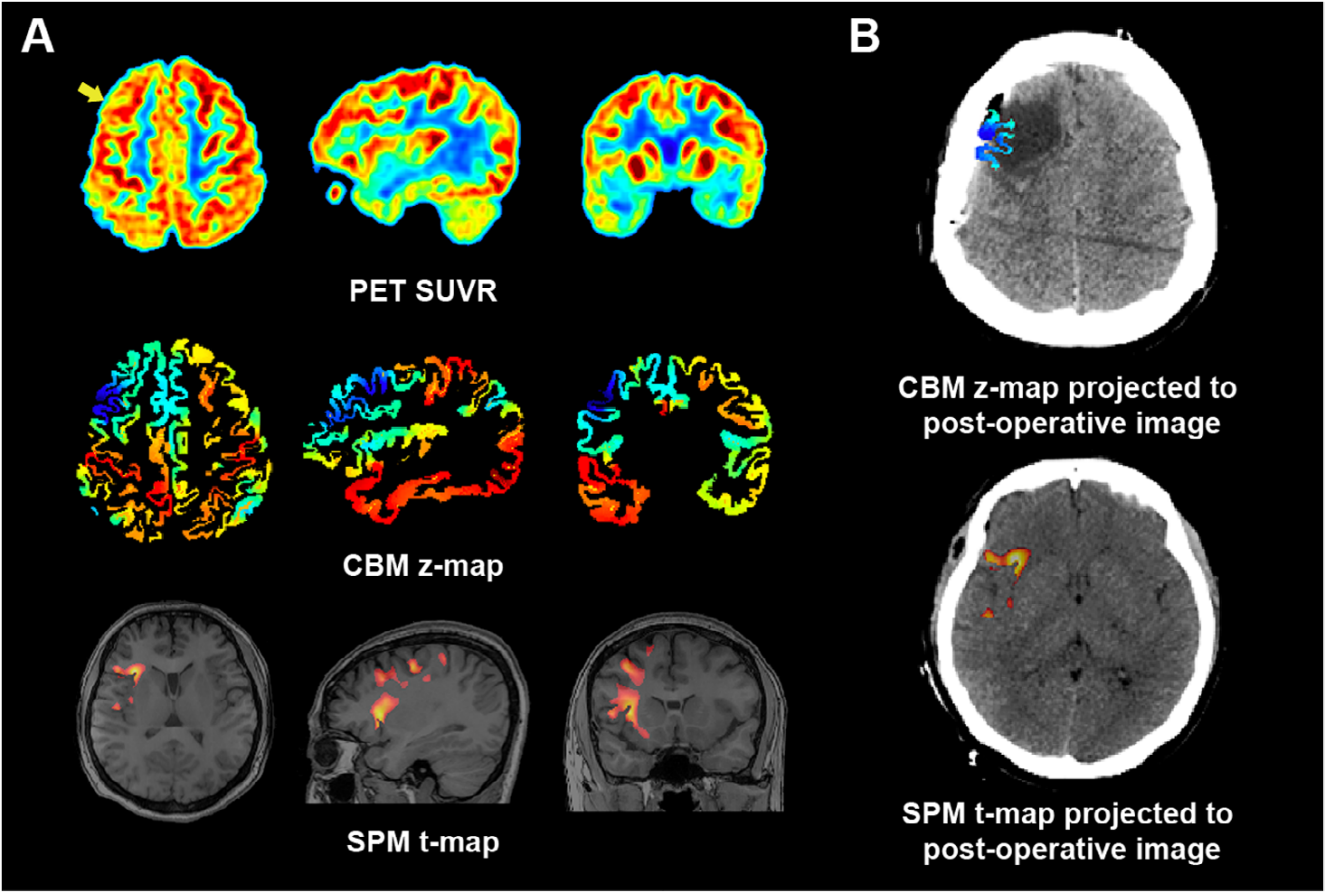
Comparisons of visual interpretations, SPM t‐maps, and CBM z‐maps with the postoperative images. No visual hypometabolism (yellow arrow) was observed at the location of the surgical field in the PET images from P023, who was seizure‐free. Clusters in the SPM t‐map were located at the anterior insular limen, whereas CBM appeared to show conclusive agreement with the surgical field.

Figure S1 shows data from five representative individuals with complex epilepsy who were seizure‐free. The epileptogenic zones in all these individuals were from functionally silent cortices, including the middle frontal gyrus, marginal sulcus, convexity of temporal‐occipital junction, insular, and inferior parietal regions. When interpreting the clinical PET images (Figure 3, Figure S2), misleading remote areas of hypometabolism outside the surgical field that could lead to potential misdiagnosis were observed. In contrast, the surgical field may even be visually normal. Regarding the t‐map clusters, false localization to the anterior insular limen was observed (Figure 3).

**FIGURE 4:**
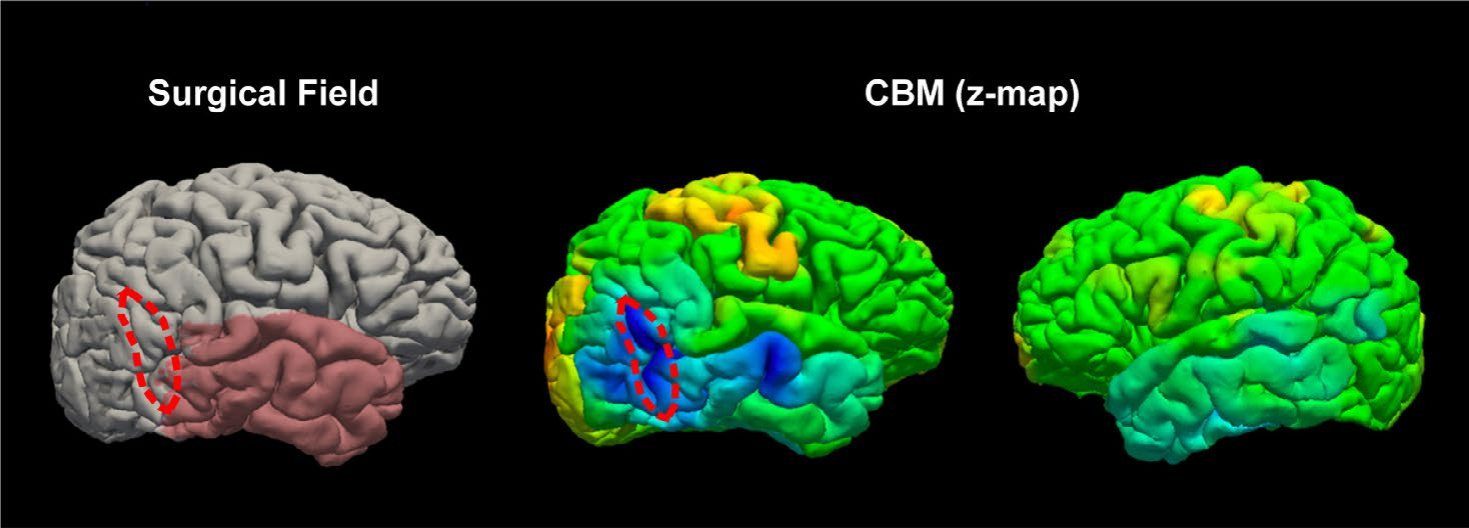
Examples of CBM z‐maps in people with worse seizure outcomes. The surgical field could not encompass all the areas with low *z* values.

Eleven people were not seizure‐free postsurgically. In six of them, CBM suggested multiple regions of hypometabolism. Data from representative individuals (Figure 4, Figure S3) are shown with hypometabolism across multiple lobes. This patient exhibited temporal semiology and ipsilateral mesial temporal hypometabolism, but anterior temporal lobectomy resulted only in an Engel‐III seizure outcome (Figure 4). The CBM of this patient suggested a potential origin outside the resected area.

### 3.4 Comparison of Concordance Between CBM and SPM Analysis

There were 12 individuals in whom CBM was conclusively concordant, whereas the SPM t‐map was partially concordant or discordant. Specifically, in two patients, the t‐maps did not reveal suggestive clusters even when a loose *p* threshold (0.05) was applied, and the global maximum *t* values were further observed. In six individuals, discordance was mainly due to artifacts resulting from edges that failed to overlap during intersubject registration. An illustrative case is shown in Figure S4. The t‐map estimated from the PET signal and the quantified artifacts of the unified morphological model (with equal gray and white matter values) exhibited a similar distribution (Figure S4). Subsequent Pearson's correlation analyses of the voxel values across the 42 individuals suggested a moderate association between the SPM t‐map and quantified artifacts (average *r*= 0.33 ± 0.02), while with CBM, there was no association with artifacts (average *r=*0.08 ± 0.02).

## 4 Discussion

Localizing seizure origin in MRI‐negative epilepsy is challenging, mainly when seizures originate from functionally silent areas [5, 6, 7]. Despite advances in electrophysiology, imaging, and postprocessing, the identification of the target for surgery with a single modality has not been achieved [11]. MRI‐negative epilepsy is often associated with subtle cortical abnormalities [23], but few studies have focused exclusively on cortical statistics to determine seizure origins. In this study, the cortex‐based method combining volumetric and surface registration approaches provides more accurate metabolic detection and uniform comparisons across subjects. With cortex‐based statistics, the CC was reached in 87.1% of the seizure‐free individuals. Notably, the CBM z‐map reliably detected seizure origins from functionally silent cortices, such as the dorsal lateral frontal, insular, and parietal lobes, and parasagittal cortices.

There were remote hypometabolic regions in people with epilepsy only, but these regions were outside the valid surgical field. These instances of hypometabolism were most likely caused by seizure propagation. Surgical resection of these two hypometabolic regions would not lead to seizure freedom. Conversely, concerning seizure origin, a decrease in ^18^F‐FDG‐PET uptake could be less obvious, and visual interpretation of the PET images in the clinical routine may lead to false positive and false negative results. There were physiological hypometabolic regions in controls. These regions are typically distributed in the limbic system and anterior temporal lobe and can appear identical to areas with seizure foci. Therefore, the temporal lobe is at a higher risk of being removed needlessly, especially for people with semiology adjacent to temporal lobe epilepsy.

As the earliest symptom of seizure onset, an aura has been suggested to provide localization information. Interpretation of aura indicates seizures adjacent to primary cortices devoted to primary functions, such as motor, sensory, visual, or auditory functions. Three‐quarters of the cerebral cortex is functionally silent. The first symptom of seizure originating from these areas is frequently a propagation symptom and usually consists of more than one overlapping symptom. No conclusive modality could suggest adequate resection when the epileptogenic zone was MRI negative. With the minimum z values in cortex‐based statistics, a detection rate of 87.1% in seizure‐free people was reached, while 83.87% of the epileptogenic zones were in functionally silent cortices, including midsagittal, frontal orbital, or insular regions. The CBM approach can identify the epileptogenic zone by finding an occult decrease while excluding physiological causes and remote propagative hypometabolism (Figure 3). As a result, the CBM method can provide more accurate information for preoperative evaluation. Thus, while the CBM approach demonstrates strong efficacy in identifying the epileptogenic zone, especially in functionally silent areas, its integration with other diagnostic methods, such as SEEG, holds great potential. This combination could further enhance the accuracy of preoperative evaluations in MRI‐negative epilepsy, ultimately improving surgical outcomes.

Classic quantification of PET images using voxelwise SPM statistics has detection rates of 40%–69%, depending on the seizure type (e.g., higher rates with temporal lobe seizures) [19, 20, 21, 31]. While cortical and subcortical information is encompassed in the SPM approaches, statistical analyses during intersubject registration (normalization) are difficult when the edges of the gyri and sulci overlap. A smoothing procedure has been proposed to solve the problem [19, 20, 21, 31], and registration artifacts can still be identified (Figure S4). With Pearson's correlation analysis, the SPM results were moderately correlated with the registration artifacts, whereas the CBM findings were not. These observations suggest the importance of adequate intersubject registration before subsequent analysis.

Common pathologies of MRI‐negative DRFE were focal cortical dysplasia, gliosis, or hamartia with gliosis [23], according to histological studies. Most of these abnormalities are in the cortical gray matter. Eliminating intersubject registration errors is critical for epilepsy. This was validated in the present study, as the classic SPM approach had a detection rate of 48.39%, compared to the cortex‐based procedures (87.1%).

There were limitations in the present study. First, as the accuracy in spatial segmentation increased, the duration for the preprocessing was prolonged to an average of 6 h in the present study. Second, this was a single‐center study with a sample size of 42 cases, which may limit the generalizability of the findings, especially when patients are subclassified into different epilepsy types. Future multicenter studies with larger samples and different scanning systems are needed to improve the method's generalizability.

## 5 Conclusions

Cortex‐based mapping from ^18^F‐FDG‐PET/MR images had a high and accurate yield in people with MRI‐negative DRFE and can be helpful for the presurgical evaluation of these patients.

## Author Contributions

G.G.Z., J.L., J.S.D., and X.H. contributed to the conception and design of the study. C.Z., Z.W., H.C., L.R., T.Y., Y.Z.S., and X.H. contributed to the acquisition and analysis of data; C.Z., Y.W., Z.W., G.G.Z., J.S.D., and J.L. contributed to drafting the text or preparing the figures.

## Ethics Statement

We confirm that we have read the Journal's position on issues involved in ethical publication and affirm that this report is consistent with those guidelines. The Ethical Committee of Xuanwu Hospital, Capital Medical University, approved this study. Written informed consent was obtained from all participants.

## Conflicts of Interest

The authors declare no conflicts of interest.

## Supporting information


**Figure S1.** Illustrative cases of 3D CBM z‐maps in 5 complex individuals. The data depict seizure origins in the middle frontal gyrus, marginal sulcus, convexity of temporal‐occipital junction, insular cortex, and inferior parietal cortex. The seizures originated from functionally silent cortices in all patients. In the first row, surgical fields were projected onto the 3D surfaces. The second row presents the minimum *z*‐scores in the CBM z‐map of the same view. The third and fourth rows show the distribution of *z* values outside the surgical field at additional angles.

**Figure S2.** Comparisons of visual interpretations, SPM t‐maps and CBM z‐maps with the postoperative images. When interpreting the clinical PET images, misleading remote areas with hypometabolism outside the surgical field (red arrows in all five) that would lead to potential misdiagnosis were observed. In contrast, the surgical field may even be visually normal (blue arrow).

**Figure S3.** Examples of CBM z‐maps in people with worse seizure outcomes. The surgical field could not encompass all the areas with low *z* values.

**Figure S4.** Illustrative case of SPM discordance and correlation analysis between SPM and CBM with quantified artifacts. CBM successfully allowed the visualization of the epileptogenic zone in P013 (with a seizure‐free outcome), but SPM images were discordant with the surgical field. Additional analyses showed that with the same postprocessing procedures, the contour image could result in similar images (voxelwise t‐map of the contour image or quantified artifacts) with the voxelwise t‐map of the PET data, while there was no visible similarity between the PET CBM z‐map and the contour CBM z‐map. Subsequent Pearson’s correlation analyses of the voxel‐to‐voxel correlation among the whole group (violin plots) indicated that the t‐maps based on PET images had a medium correlation with the quantified artifacts, while the CBM z‐maps showed no correlation.

**Table S1.** Seizure outcomes and diagnostic performance.

## Data Availability

The data that support the findings of this study are available from the corresponding author upon reasonable request.
