# Supplementary figures and images for: Quantitative Cortex‐Based Mapping With Hybrid 18F‐FDG‐PET/MR Images in MRI‐Negative Epilepsy
Source: CNS Neurosci Ther. 2025 Apr 18;31(4):e70336. doi: 10.1111/cns.70336 (PMC12008173; doi:10.1111/cns.70336)

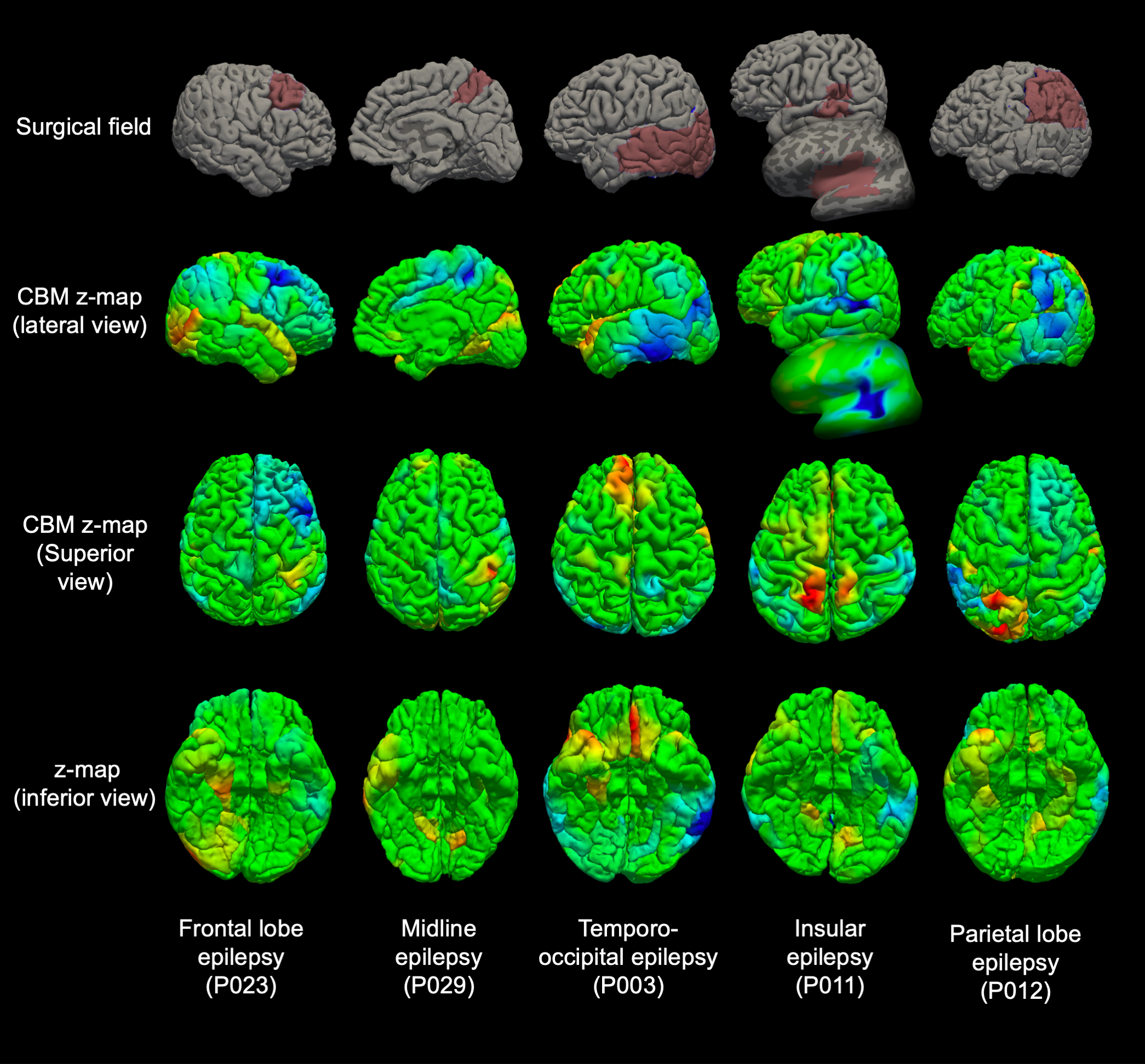

Supplement: Supplementary file 1 — Figure S1. Illustrative cases of 3D CBM z‐maps in 5 complex individuals. The data depict seizure origins in the middle frontal gyrus, marginal sulcus, convexity of temporal‐occipital junction, insular cortex, and inferior parietal cortex. The seizures originated from functionally silent cortices in all patients. In the first row, surgical fields were projected onto the 3D surfaces. The second row presents the minimum z‐scores in the CBM z‐map of the same view. The third and fourth rows show the distribution of z values outside the surgical field at additional angles. [file CNS-31-e70336-s002.tif]

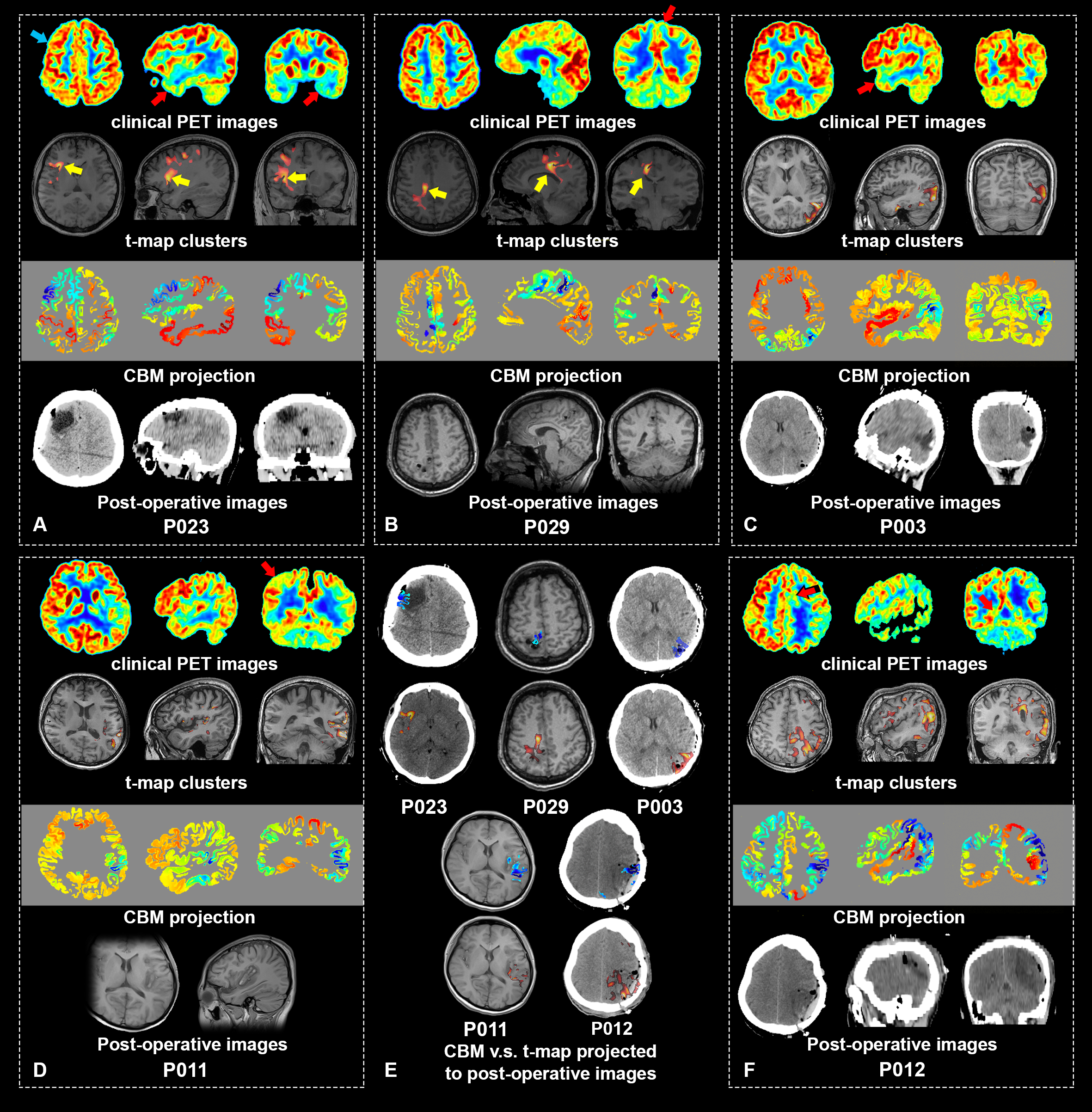

Supplement: Supplementary file 2 — Figure S2. Comparisons of visual interpretations, SPM t‐maps and CBM z‐maps with the postoperative images. When interpreting the clinical PET images, misleading remote areas with hypometabolism outside the surgical field (red arrows in all five) that would lead to potential misdiagnosis were observed. In contrast, the surgical field may even be visually normal (blue arrow). [file CNS-31-e70336-s005.tif]

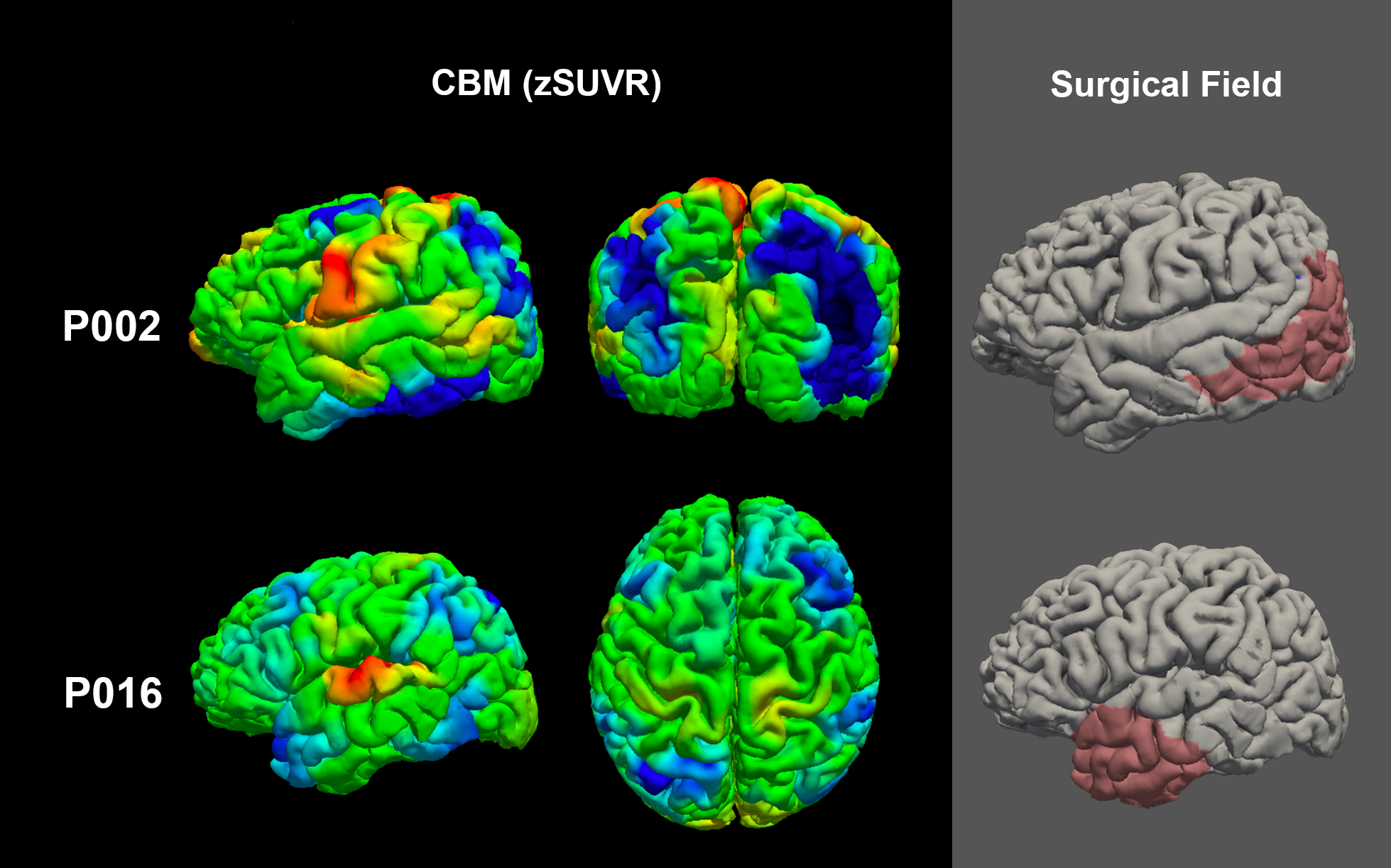

Supplement: Supplementary file 3 — Figure S3. Examples of CBM z‐maps in people with worse seizure outcomes. The surgical field could not encompass all the areas with low z values. [file CNS-31-e70336-s001.tif]

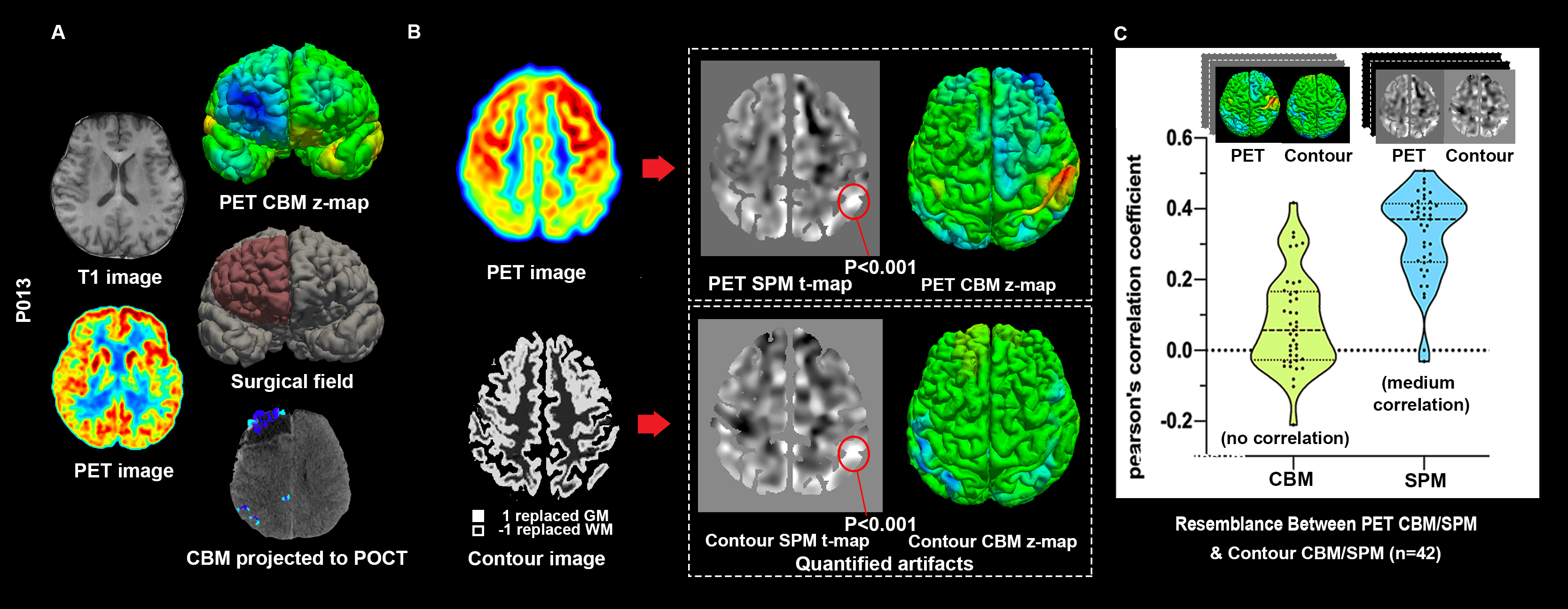

Supplement: Supplementary file 4 — Figure S4. Illustrative case of SPM discordance and correlation analysis between SPM and CBM with quantified artifacts. CBM successfully allowed the visualization of the epileptogenic zone in P013 (with a seizure‐free outcome), but SPM images were discordant with the surgical field. Additional analyses showed that with the same postprocessing procedures, the contour image could result in similar images (voxelwise t‐map of the contour image or quantified artifacts) with the voxelwise t‐map of the PET data, while there was no visible similarity between the PET CBM z‐map and the contour CBM z‐map. Subsequent Pearson’s correlation analyses of the voxel‐to‐voxel correlation among the whole group (violin plots) indicated that the t‐maps based on PET images had a medium correlation with the quantified artifacts, while the CBM z‐maps showed no correlation. [file CNS-31-e70336-s004.tif]
