# Supplementary material for: Quantitative Cortex‐Based Mapping With Hybrid 18F‐FDG‐PET/MR Images in MRI‐Negative Epilepsy
Source: CNS Neurosci Ther. 2025 Apr 18;31(4):e70336. doi: 10.1111/cns.70336 (PMC12008173; doi:10.1111/cns.70336)
Supplement: Supplementary file 5 — Table S1. Seizure outcomes and diagnostic performance. [file CNS-31-e70336-s003.docx]

| **Supporting Table 1 Seizure outcomes and diagnostic performance** | | | | | | | | | | | |  |
| --- | --- | --- | --- | --- | --- | --- | --- | --- | --- | --- | --- | --- |
| Individual ID | CBM z-map Rating | SPM t-map rating | SUVR Rating | Sides | | | | Location | | | | |
|  |  |  |  | Surgical Field | CBM z-map | SPM t-map | SUVR | Surgical Field | CBM z-map | SPM t-map | SUVR (visual) | |
| P001 | 1 | 1 | 3 | L | L | L | B | MFG | MFG | L:MFG (R:OL） | TL | |
| P002 | - | - | - | L | B | B | B | TPO | L:TPO,MFG, R:TPO | L:TPO,MFG, R:TPO | L:TPO,MFG,TL, R:TPO,TL | |
| P003 | 1 | 1 | 3 | L | L | L | L | TPO | TPO | TPO | MTL | |
| P004 | 1 | 3(max t-values) | 2 | R | R | B | B | ACG | ACG | OFC | LS | |
| P005 | 1 | 3 | 2 | R | R | L | B | STG | STG | PL | TL | |
| P006 | 1 | 1 | 2 | L | L | L | B | S/MFG | S/MFG | S/MFG | L:MFG,MTL,OL, R:MFG | |
| P007 | 1 | 1 | 3 | R | R | R | B | Insula | PI | PI | R: PL, B: MTL | |
| P008 | 3 | 3 | 3 | L | B | L | B | SPL | R: MFL. L: ATL | ATL | TL | |
| P009 | - | - | - | R | B | B | B | PL | IPL | PL | TL | |
| P010 | - | - | - | L | L | L | B | S/MFG | MFG, PL | MFL | L: OFC. B: MTL | |
| P011 | 1 | 1 | 2 | L | L | L | B | Insula, STG | Insula, STG | Insula, STG | TL | |
| P012 | 1 | 1 | 2 | L | L | L | B | PL | IPL | IPL | L: IPL, TL. R: MTL | |
| P013 | 1 | 3 | 2 | R | R | B | B | FP | FP | PL | R: FL,TL. L:MTL | |
| P014 | 1 | 1 | 3 | L | L | L | B | PTL | PTL | PTL, PL | MTL | |
| P015 | - | - | - | R | B | R | B | MCG | L:PL, R:MCG | MCG | R: ACG. B: ATL | |
| P016 | - | - | - | L | R | L | R | ATL | IPL | TPO | HP&AM | |
| P017 | 1 | 3 | 3 | L | L | L | B | RO | RO | PL | MTL | |
| P018 | - | - | - | L | B | L | B | RO | RO | L:RO,PL | B: ATL, RO. L: IPL | |
| P019 | - | - | - | R | R | R | B | ATL | TPO, TL | TPO | B: TL, R:TPO, OFC | |
| P020 | 1 | 1 | 2 | L | L | L | B | ATL | ATL | ATL | ATL | |
| P021 | 1 | 1 | 2 | L | L | L | B | MFL | MFL | MFL(PL) | B: MFL, ATL | |
| P022 | 1 | 1 | 3 | L | L | L | B | FL | FL | FL | MTL | |
| P023 | 1 | 3 | 3 | R | R | R | B | S/MFG | MFG | Insula | MTL | |
| P024 | 1 | 3 | 3 | R | R | R | R | S/MFG | MFG | PL | TL, ACG | |
| P025 | - | - | - | R | B | R | L | MFL | L: FL, R: ACG | ACG | RR, ATL | |
| P026 | 1 | 3 | 3 | R | R | L | B | RR | RR | RR | MTL | |
| P027 | 1 | 3 | 2 | R | R | L | B | OFC | OFC | ATL | R: OFC, FP. L: FPL | |
| P028 | - | - | - | L | B | L | B | PL | L:MPL, R:PL | PL | L: PL, TL, R: PL | |
| P029 | 1 | 3 | 2 | R | R | R | B | MS | MS | MCG | R: MS, ACG. B: MTL | |
| P030 | 1 | 3 | 2 | R | R | L | B | FO, RR, Insula | FO, RR, Insula | FO | R: FO. B: MTL | |
| P031 | 1 | 1 | 2 | L | L | L | B | T-Neocortex | T-Neocortex | ATL | L: TL. R: MTL | |
| P032 | 1 | 1 | 2 | R | R | R | B | T-Neocortex | T-Neocortex | ATL | R: TL. L: MTL | |
| P033 | 1 | 1 | 2 | R | R | R | B | MFG | MFG | MFG | R: FL, L: TL | |
| P034 | 2 | 2 | 2 | R | R | R | B | TL | TL | ATL, PL | ATL | |
| P035 | - | - | - | R | R | R | B | ATL | TPO | ATL | R:ATL, PL, L: HP&AM | |
| P036 | 3 | 3 | 3 | L | R | R | B | FL | PCG | ATL | R: PCG, L: TP | |
| P037 | 1 | 1 | 2 | R | R | R | B | FP | TP | ATL | R:ATL, PL, L: HP&AM | |
| P038 | 3 | 3 | 3 | L | L | L | B | TPO | T-Neocortex | ATL | FL, TL | |
| P039 | 1 | 1 | 2 | L | L | B | B | TL | T-Neocortex | TL | FL,TL | |
| P040 | 1 | 3 | 3 | R | R | L | B | MFG | MFG | ATL | ATL | |
| P041 | 1 | 2(max t values) | 3 | R | R | R | B | FL&PL | FO,TO | AM | HP&AM | |
| P042 | - | - | - | L | L | L | L | PL | IPL | HP | IPL | |
| L: left; R: right; B: bilateral; MFG: Middle frontal gyrus; TL: Temporal lobe; TPO: Temporo-parieto-occipital junction; MTL: Mesial temporal lobe; ACG: Anterior cingulate gyrus; LS: Limbic system; TP: temporal pole; SFS: Superior frontal sulcus; PI: posterior insular; IPL: inferior parietal lobe; OFC: Orbital frontal cortex; STG: Superior temporal gyrus; PL: Parietal lobe; SPL: Superior parietal lobe; MFL: Mesial frontal lobe; FP: Frontal pole; FL: Frontal lobe; PTL: Posterior temporal lobe; STS: Superior temporal sulcus; MCG: Middle cingulate gyrus; RO: Rolandic operculum; RR: Rolandic region; MS: Marginal sulcus; FO: Frontal operculum; PCG: Posterior cingulum gyrus; TO: temporal operculum; T-Neocortex: temporal neocortex; HP: Hippocampus; AM: Amygdala; OL: Occipital lobe；1：Conclusive concordant; 2: Partial concordant; 3: Discordant | | | | | | | | | | | |  |
